# Supplementary material for: Impact of Cytomegalovirus Infection and Genetic Background on the Frequencies of Peripheral Blood Suppressor Cells in Human Twins
Source: Pathogens. 2021 Jul 30;10(8):963. doi: 10.3390/pathogens10080963 (PMC8399020; doi:10.3390/pathogens10080963)
Supplement: Supplementary file 1 [file pathogens-10-00963-s001.zip › pathogens-1275376-supplementary.pdf]

pathogens

MDPI

Table S1. Characteristics of the study participants; 81 twin pairs, MZ (Monozygotic), DZ (same-sex Dizygotic).

	All	Concordant CMV Seronegative		Concordant CMV Seropositive		CMV Discordant	
		MZ	DZ	MZ	DZ	MZ	DZ
Pairs	81	8	8	23	21	11	10
Gender[1]	124/38	7F/1M	7F/1M	16F/7M	17F/4M	8F/3M	7F/3M
Median age in years	48	44.5	47	48	49	44	58
Age range	41–77	42–47	44–72	41–70	41–77	41–59	43–73

[1] F= female, M = male.

Table S2. Impact of CMV-serostatus, age and gender on immune phenotypes.

Immune Subset	CMV- [1]	CMV+ [1]	≤48 [2]	>48 [2]	Female	Male
CD25+	2.9%	2.5% *	2.7%	2.4%	2.5%	3.0%
FoxP3+	3.6%	3.1%	3.1%	3.7% *	3.1%	3.6%
CD25+FoxP3+	2.9%	2.4%	2.4%	2.7% *	2.5%	2.5%
Non suppressive Tregs	1.9%	1.6%	1.7%	1.7%	1.7%	1.5%
CD25+FoxP3+Helios+	1.7%	1.4%	1.3%	1.7% **	1.4%	1.7%
resting Tregs	0.5%	0.5%	0.5%	0.5%	0.5%	0.4%
activated Tregs	2.1%	1.8%	1.8%	1.9%	1.8%	2.0%
activated CD25+ Tregs	1.7%	1.5%	1.5%	1.7%	1.5%	1.8%
FoxP3-Helios+	2.1%	2.0%	2.0%	2.0%	2.0%	2.3%
FoxP3+Helios+	2.0%	1.7%	1.6%	2.1% **	1.7%	2.0%
FoxP3+Helios-	1.8%	1.5% *	1.6%	1.7%	1.6%	1.5%
CD45RA+CD45RO-	41.0%	37.8%	38.4%	38.4%	38.2%	38.8%
CD45RA+CD45RO-FoxP3+Helios+	0.05%	0.05%	0.04%	0.07% ***	0.05%	0.05%
CD45RA-CD45RO+FoxP3+Helios+	1.5%	1.4%	1.4%	1.6%	1.4%	1.8%
CD8+FoxP3+	0.5%	0.4%	0.5%	0.6%	0.5%	0.4%
CD8+Helios+	21.5%	17.0%	18.4%	16.4%	18.9%	12.7%
CD14+	14.9%	12.1%	12.2%	12.4%	12.2%	12.4%
Lin-	32.1%	26.8% *	27.4%	28.9%	28.0%	30.0%
Lin-CD14-	16.0%	14.4%	14.2%	16.1%	14.5%	15.4%
Lin-CD14-CD15+	0.07%	0.06%	0.05%	0.06%	0.06%	0.07%
Lin-CD14-CD15+CD11b+	0.06%	0.05%	0.06%	0.05%	0.05%	0.06%
Lin-CD16-CD14+	14.1%	10.8%	11.2%	11.5%	11.3%	11.5%
Lin-CD16-CD14+HLA-DR+	11.0%	9.4%	9.7%	9.9%	9.9%	9.9%
Lin-CD16+CD14+	0.9%	0.8%	0.6%	0.9% *	0.9%	0.6%
Lin-CD16+CD14+HLA-DR+	0.8%	0.8%	0.6%	0.9% *	0.8%	0.6%
Lin-CD16+CD14low	9.9%	8.7%	7.9%	11.8% *	9.0%	8.8%
Lin-CD16+CD14lowHLA-DR+	1.5%	1.6%	1.1%	2.0% **	1.5%	1.5%
Lin-CD16-CD14-	5.3%	4.9%	5.4%	4.5%	4.7%	5.8% *
Lin-CD14+HLA-DR-	1.4%	0.5% **	0.8%	0.9%	0.8%	0.4%
Lin-CD14+HLA-DR+	11.7%	10.0%	10.3%	10.9%	10.4%	11.0%
Lin-CD14-HLA-DR-	3.9%	3.5%	3.4%	3.9%	3.7%	3.1%
Lin-CD14-HLA-DR+	12.3%	11.0%	10.5%	12.2%	10.9%	12.6% *

[1] CMV- = CMV-seronegative, CMV+ = CMV-seropositive. [2] 48 years is the median, median frequencies displayed. Mann Whitney p values with * $p \leq 0.05$, ** $p < 0.01$, *** $p < 0.001$.

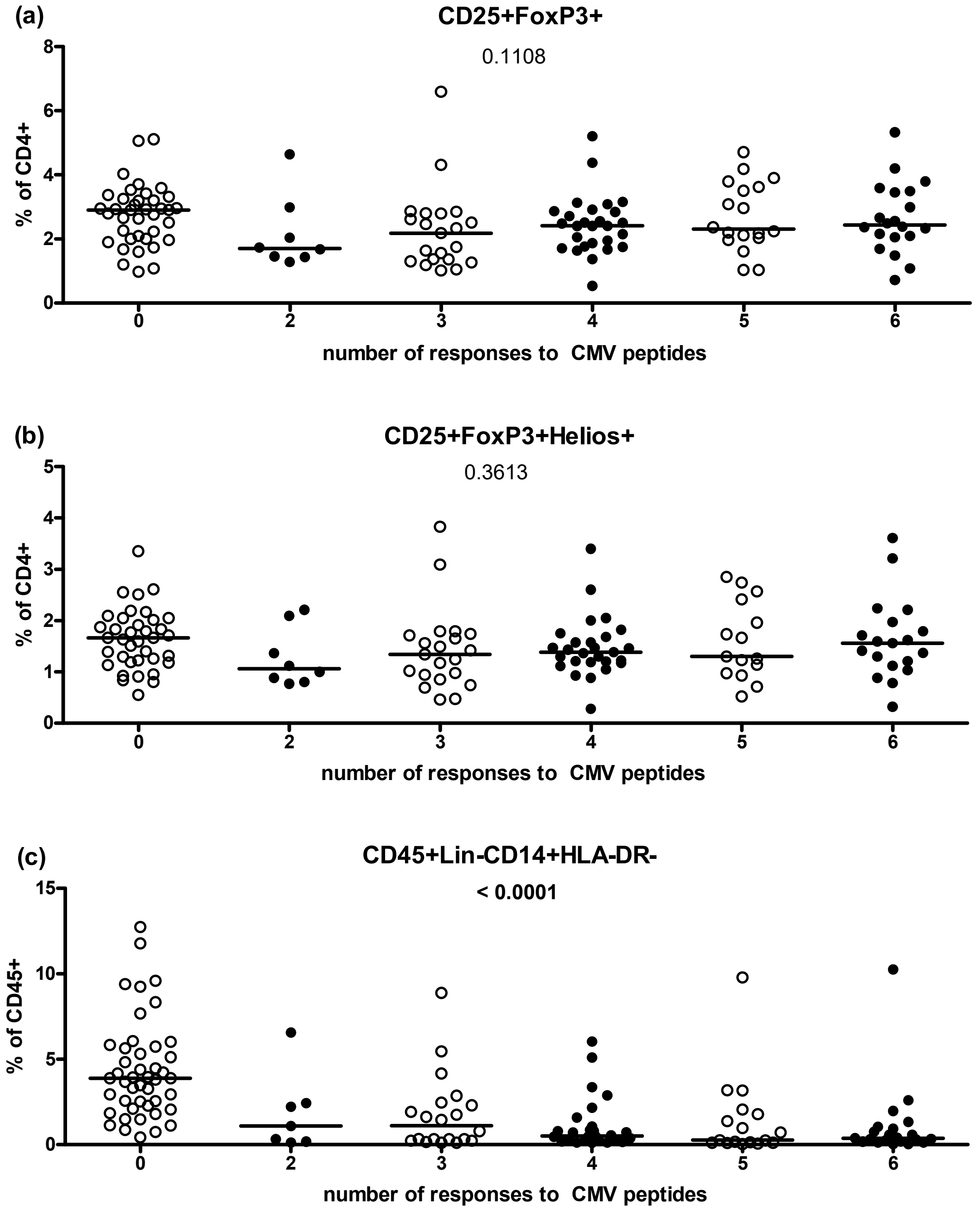

Figure S1. Frequencies of CD25+FoxP3+ (**a**), CD25+FoxP3+Helios+ (**b**) and CD14+HLA-DR- (**c**) suppressor cells within CMV-seronegative and CMV-seropositive twins grouped according to the number reactivities to the 6 CMV peptides investigated (IE-1, p150, CM2, p65, gB-1, gB-2). Borderline responses were considered negative. *P* values are shown for Kruskal-Wallis test and horizontal bars represent the medians.

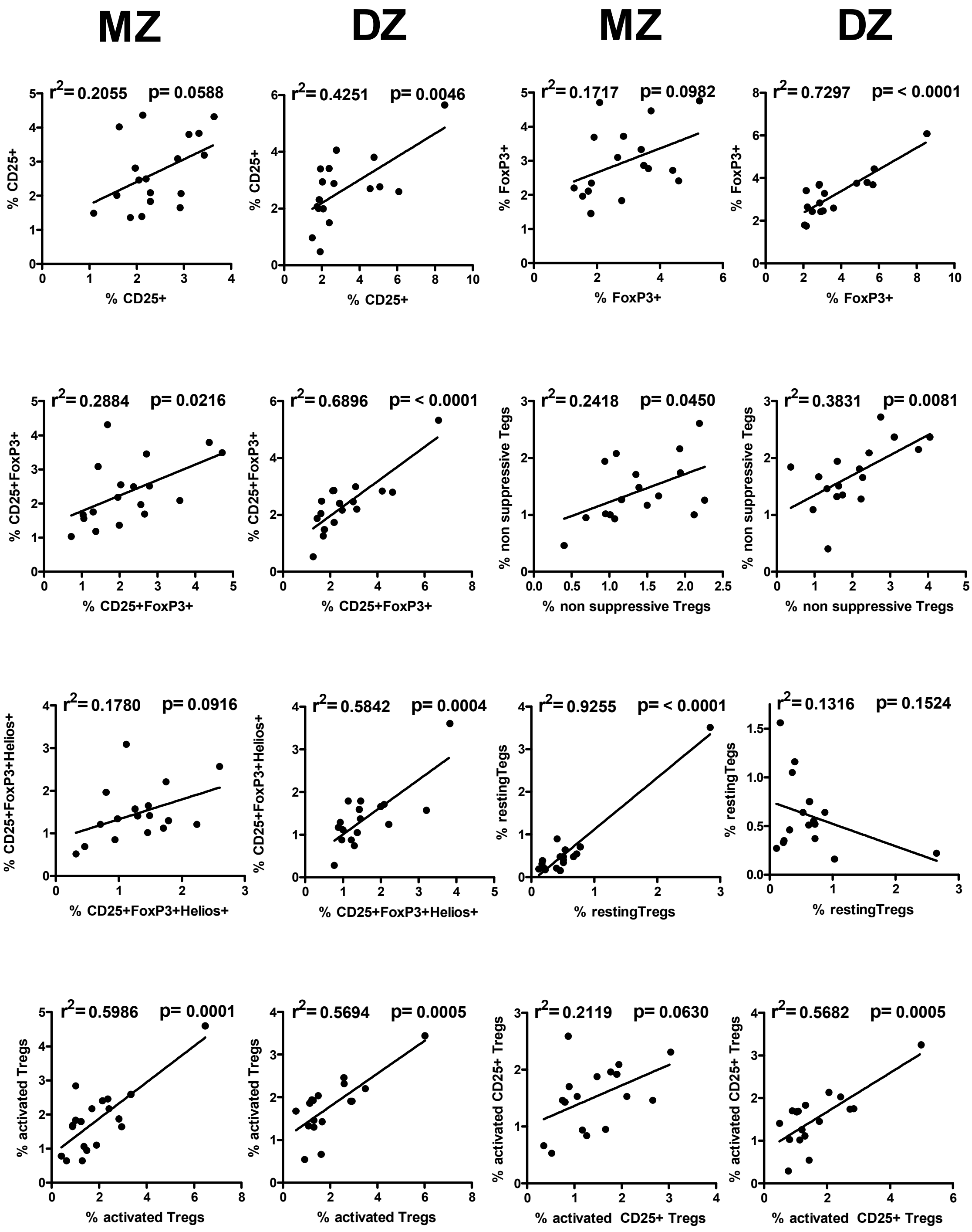

Figure S2. Correlation analysis for differentiation status of Tregs within CMV-seropositive twin pairs. Scatter plots are shown for CMV-seropositive concordant monozygotic (MZ) and dizygotic (DZ) pairs. Each symbol represents one twin pair.

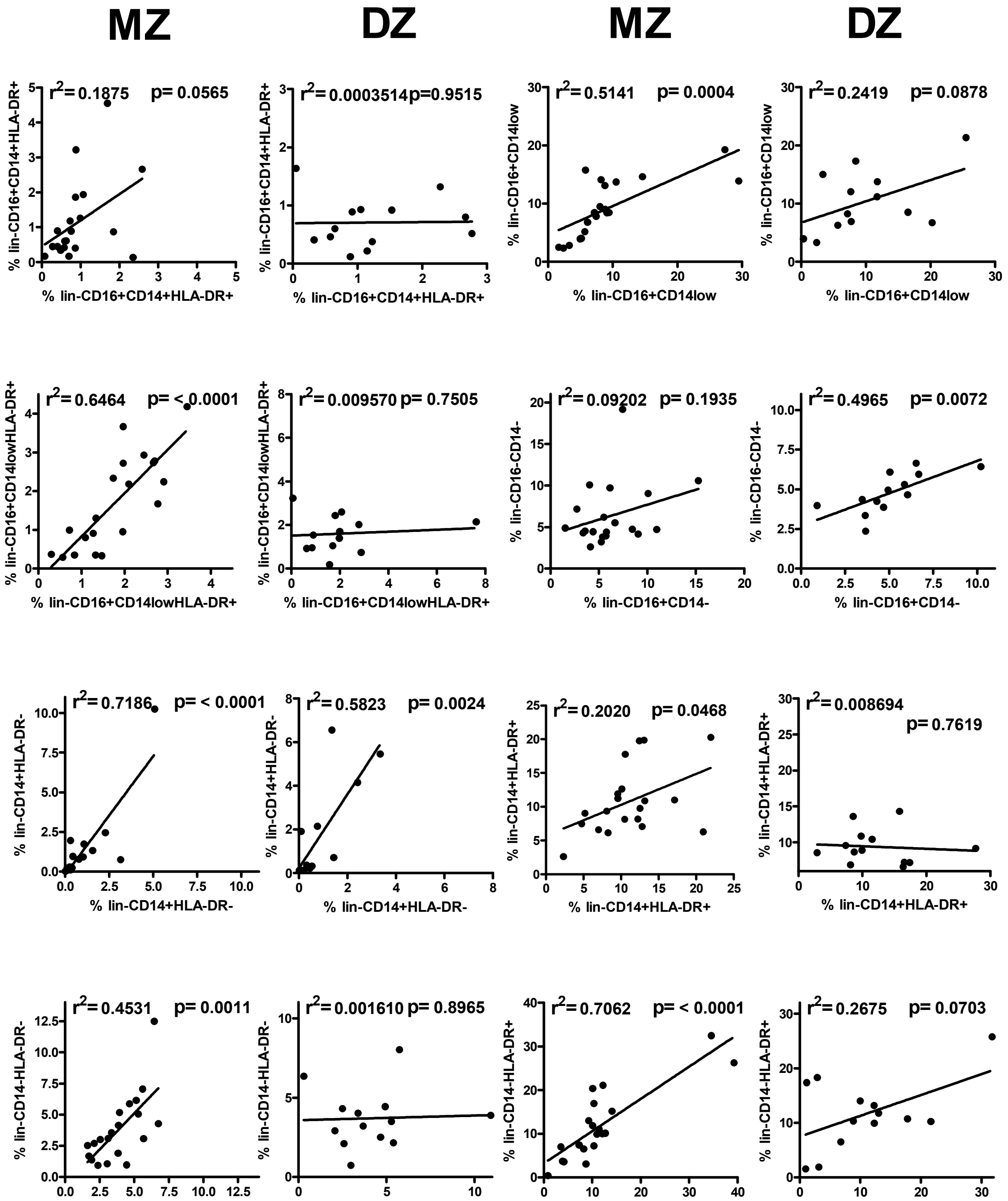

Figure S3. Correlation analysis for differentiation status of monocytes and MDSCs between CMV-seropositive co-twins. Scatter plots are shown for CMV-seropositive concordant monozygotic (MZ) and dizygotic (DZ) pairs. Each symbol represents one pair.

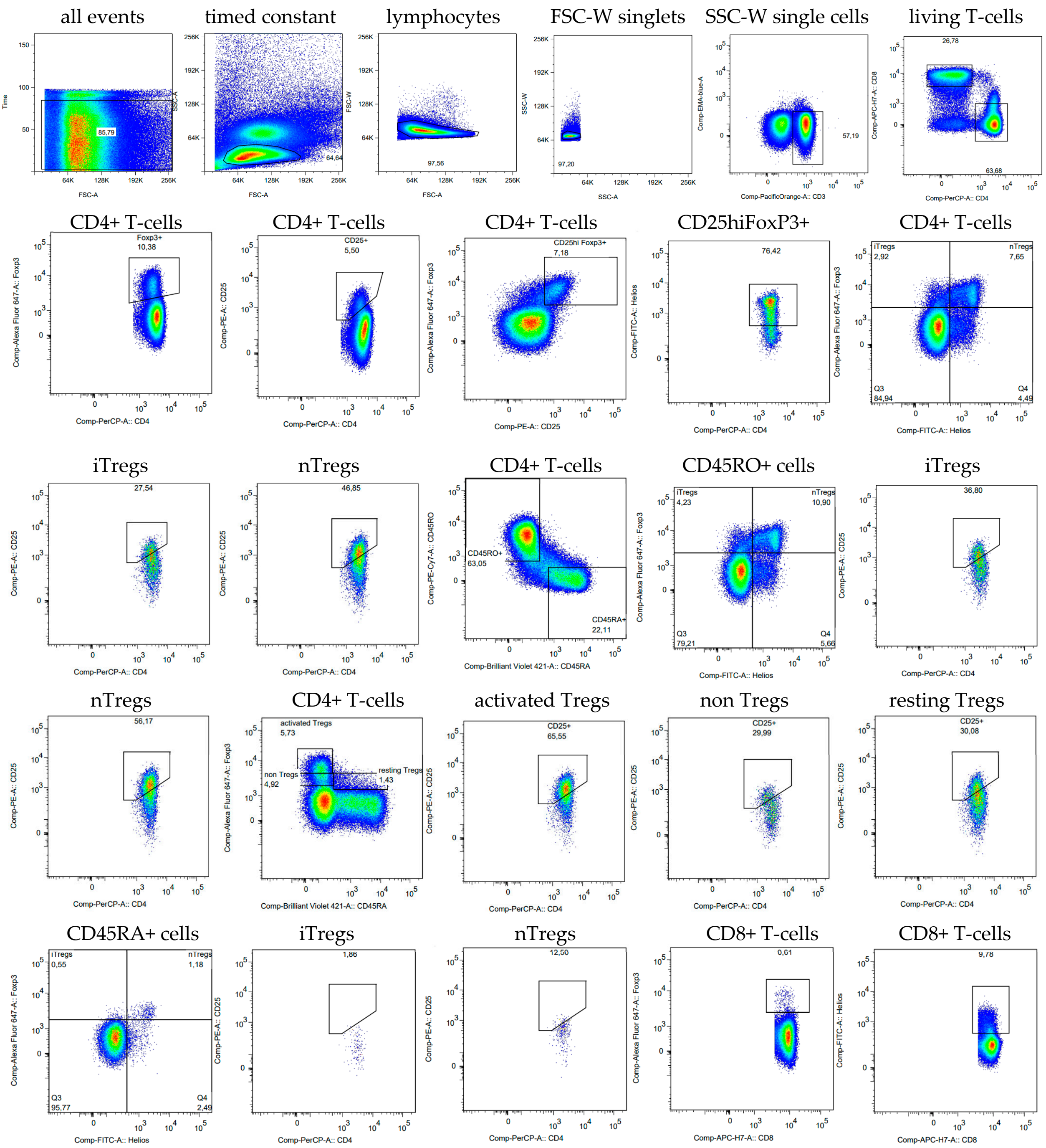

Figure S4. Treg panel gating strategy. Starting with all measured events various Treg phenotypes have been gated with the parental population named above each plot, here for a control sample.
